# Cutaneous Metastasis of Medullary Carcinoma Thyroid Masquerading as Subcutaneous Nodules Anterior Chest and Mandibular Region

**DOI:** 10.1155/2014/805205

**Published:** 2014-11-11

**Authors:** Rahul Mannan, Jasmine Kaur, Jasleen Kaur, Sanjay Piplani, Harjot Kaur, Harleen Kaur

**Affiliations:** ^1^Department of Pathology, SGRDIMSR, Amritsar, Punjab 143001, India; ^2^Department of Oral and Maxillofacial Surgery, SGRDIMSR, Amritsar, Punjab, India; ^3^Department of Dermatology, SGRDIMSR, Amritsar, Punjab, India

## Abstract

Cutaneous metastasis of underlying primary malignancies can present to dermatologist with chief complaints of cutaneous lesions. The underlying malignancy is generally diagnosed much later after a complete assessment of the concerned case. Medullary carcinoma thyroid (MCT) is a relatively uncommon primary neoplasia of the thyroid. Very few cases presenting as cutaneous metastases of MCT have been reported in the literature. Most of the cases which have been reported are of the papillary and the follicular types. We here report a case of a patient who presented in the dermatology clinic with the primary complaint of multiple subcutaneous nodules in anterior chest wall and left side of body of mandible. By systematic application of clinical and diagnostic skills these nodules were diagnosed as cutaneous metastasis of MCT bringing to the forefront a history of previously operated thyroid neoplasm. So clinically, the investigation of a flesh coloured subcutaneous nodule, presenting with a short duration, particularly in scalp, jaw, or anterior chest wall should include possibility of metastastic deposits. A dermatologist should keep a possibility of an internal organ malignancy in patients while investigating a case of flesh coloured subcutaneous nodules, presenting with short duration. A systematic application of clinical and diagnostic skills will eventually lead to such a diagnosis even when not suspected clinically at its primary presentation. A prompt and an emphatic diagnosis and treatment will have its bearing on the eventual outcome in all these patients.

## 1. Introduction

Organ specific malignancies rarely present clinically as cutaneous metastasis. Such patients often report to a dermatologist with the chief complaint of cutaneous lesions. The underlying malignancy is generally diagnosed after a complete assessment of the concerned case [1]. Medullary carcinoma thyroid (MCT) is a relatively uncommon primary neoplasia of the thyroid. Very few cases presenting as cutaneous metastases of MCT have been reported in the literature. Most of the cases which have been reported are of the papillary and the follicular types with the most frequent site of presentation being scalp [2].

We report a case of a patient who presented in the dermatology clinic with the primary complaint of multiple subcutaneous nodules in anterior chest wall and left side of body of mandible. These nodules were diagnosed as cutaneous metastasis of MCT on cytology bringing to the forefront a history of previously operated thyroid neoplasm.

## 2. Case Report

**Figure 1:**
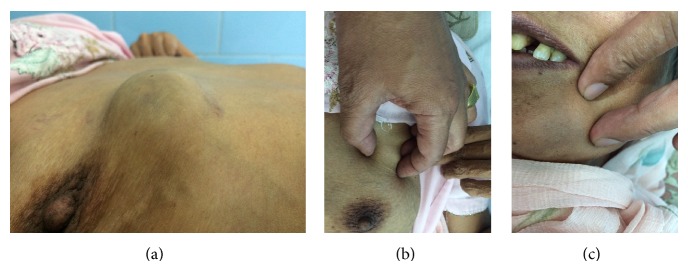
(a) Subcutaneous nodule seen in the anterior chest wall below the right breast. (b) Another subcutaneous nodule seen in the anterior chest wall just near the left breast. (c) Subcutaneous nodule in the mandible.

A 43-year-old female patient presented in the dermatology clinic of a tertiary care teaching hospital with primary complaint of gradually increasing multiple painless swellings on the anterior chest wall and in the jaw for the past 3 months. On examination, subcutaneous nodules were observed and their size ranged from the smallest being 1.0 cm (left side mandible) to the largest which measured 3.0 cm (below the right breast) with the overlying skin being normal in colour (Figures 1(a), 1(b), and 1(c)). On palpation these nodules were nontender with no temperature elevation, nonitchy, soft to firm in consistency, freely mobile, and not fixed to the underlying tissues.

General clinical examination of the patient was nonsignificant. The various routine haematological parameters were within normal limits with no eosinophilia or atypical cell noted in peripheral smear examination. Her biochemical tests were within range and serological investigations noncontributory (nonreactive viral markers including negative VDRL serology). On the basis of clinical picture, lab investigations and symptomatology the clinical differentials of lipomatous lesion, benign neural sheath tumour, fibroblastic/fibrohistiocytic lesion, adnexal tumour, and amelanotic melanoma were made.

To ascertain the etiology of the nodules, fine needle aspiration cytology (FNAC) was planned under the guidance of dermatologist in order to aspirate the material from correct representative site. The procedure was done from multiple sites with the help of 23 G needles and 2-3 passes were given. Material aspirated was serosanguinous. Smears prepared were air dried and alcohol fixed. May grunwald giemsa (MGG) and hematoxylin and eosin (H & E) stains were done, respectively. Extra blood admixed material was taken and cell block was made which was sent to histopathology unit for tissue processing.

**Figure 2:**
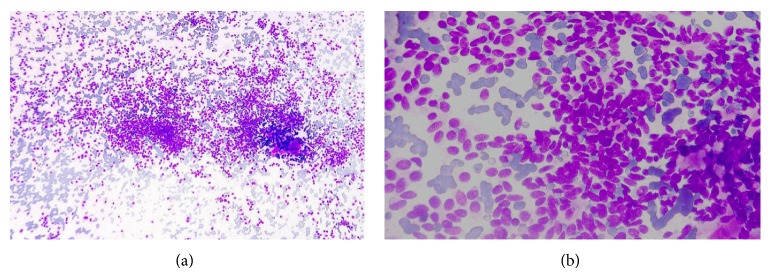
(a) Singly scattered cellular aspirate with cells of variable sizes and shapes on fine needle aspiration [MGG ×100]. (b) Higher magnification exhibiting predominantly oval and spindloid to plasmacytoid cells on fine needle aspiration [H & E 400x].

The smears prepared showed dispersed cellular aspirate with cells of variable sizes and shapes. The cells were predominantly oval and spindloid to plasmacytoid. The nuclei of these cells exhibited mild anisokaryosis, presence of small inconspicuous nucleoli, and speckled (salt and pepper) chromatin (Figures 2(a) and 2(b)). The cytoplasm of these cells was eosinophilic and demonstrated fine, pinkish cytoplasmic granularity. Focally, presence of small quantity of amorphous pinkish material (amyloid like material) was also observed.

The cytological opinion was thus in favour of subcutaneous deposits of a “neuroendocrinal” lesion. In context of the cytological findings the patient was reevaluated at the dermatological clinic for the same. During reevaluation the patient concurred with the symptoms associated with neuroendocrinal lesions of intermittent episodes of flushing. Based on these findings a provisional diagnosis of a neuroendocrinal lesion arising from the gastrointestinal tract, lungs or thyroid was made. The patient was asked about history of any previous surgery in relation to the abovementioned sites. The patient subsequently provided a history of thyroid surgery 5 years back at another centre. On examination a fine scar was noted in one of the neck creases. The previous treatment records and reports were not available with the patient which is quite the norm in many instances in resource challenged countries. Based on these findings, a working diagnosis of cutaneous metastatic deposits of MTC was suggested with histopathology report of cell block awaited.

**Figure 3:**
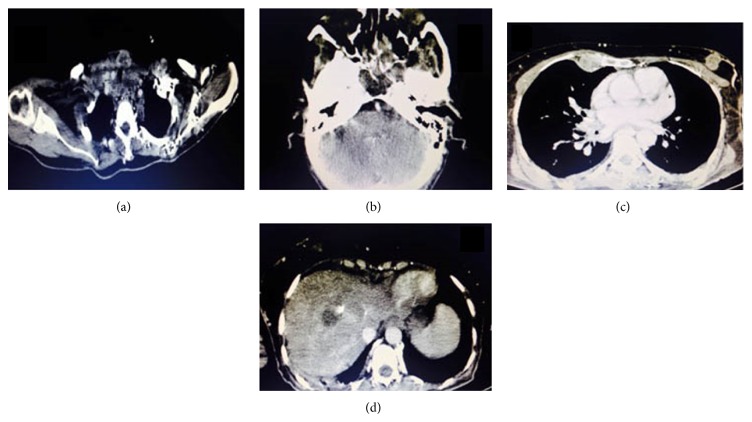
(a) Destruction of the right 4th rib along with erosion of the spinous processes of the thoracic vertebra and sclerotic lesions in the body of the vertebrae. (b) Destruction of the floor of middle cranial fossa, posterior ethmoidal air cells and sphenoid sinus. (c) Lesions in the mediastinum. (d) In liver multiple variable sized heterogeneous lesions containing foci of calcification were observed.

Patient was advised radiological examination (CT scan and ultrasonography). CT scan revealed destruction of the floor of middle cranial fossa, posterior ethmoidal air cells, and sphenoid sinus. In the maxillofacial region there was destruction of the left condylar process of mandible with soft tissue mass extending into the infratemporal fossa. Destruction of the left alveolar margin of the mandible was also present. CT scan of the neck region showed a heterogeneously enhanced nodular mass in the residual thyroid gland with retrosternal extension. Multiple lymph nodes were seen at level II, III, and supraclavicular region bilaterally. In the thoracic region, three irregular nodular spiculated masses with central necrosis were seen in the right breast parenchyma. Destruction of the right 4th rib along with erosion of the spinous processes of the thoracic vertebra and sclerotic lesions in the body of the vertebrae were noted. In liver multiple variable sized heterogeneous lesions containing foci of calcification were observed. These were also noted as multiple hyperechoic lesions in both lobes of liver on USG (suggestive of metastatic deposits) (Figure 3).

**Figure 4:**
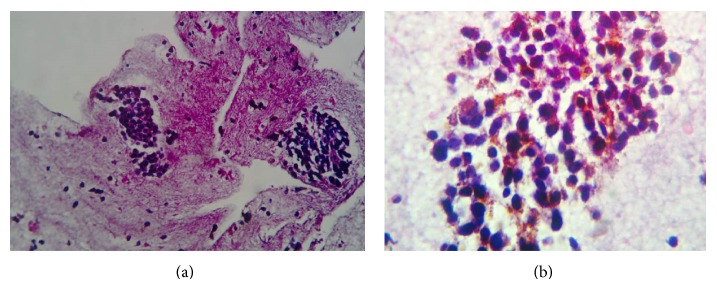
(a) Small nests of cohesive malignant cells within areas of hemorrhage [H & E 100x]. (b) Higher magnification detailing the cell morphology of spindle to oval shaped cells [H & E 400x].

The histopathology report of the cell block showed a lesion composed of nests of cohesive malignant cells within areas of hemorrhage (Figure 4(a)). The individual cells were predominantly spindle shaped to epithelioid in morphology. They contained a moderate amount of amphophilic cytoplasm with irregular, hyperchromatic nuclei containing prominent nucleoli exhibiting characteristic “salt and pepper” chromatin. The cytoplasm was minimal but showed fine eosinophilic granules (Figure 4(b)).

On immunohistochemical studies (IHC), tumor cells were positive for calcitonin, TTF-1, and thyroglobulin thus confirming the primary site to be thyroid and the tumor. Hence a final diagnosis of cutaneous metastasis of MTC was rendered. Patient was advised whole body scan, calcitonin and CEA serum assays for further evaluation and also offered chemotherapy and palliative radiotherapy which the patient refused due to economic constraints. Patient and her attendants were advised and encouraged to undergo a full clinical/radiological examination and screening for germ line mutation. Patient was also unable to give a proper family history or presence of any relative with such manifestations/thyroid swelling.

For treatment purposes she was referred to a higher centrally funded apex centre where she could receive adequate subsidized therapy for the same.

## 3. Discussion

Subcutaneous nodules can cause a dilemma to both the treating physician (in this case dermatologist) and the cytopathologist to reach a conclusive diagnosis. This is more so pronounced in reaching a cytodiagnosis of cutaneous metastasis in the absence of history of primary tumour as in the present case. In cutaneous metastasis of MCT, the cytology and the clinical opinion can be biased towards a diagnosis of adnexal tumour/fibrous lesion (due to presence of spindloid cells of MCT). So a caution has to be exercised.

Skin metastasis of thyroid cancer has been rarely reported and of which most of the cases documented are of papillary and follicular type [3] with MCT being the least common with around 10 cases reported worldwide. The most common site of cutaneous metastasis of thyroid neoplasia reported in the literature is scalp [4].

In the present case report, a point of difference from the other case reports detailing the cutaneous metastasis of MCT was localization (absence of scalp lesions and presence of nodule in mandible region) and presentation (as the nodules were skin coloured, nonitching, and nonulcerative) and associated with no granulation tissue and hyperkeratinization.

MCT is a variant of thyroid carcinoma which originates from the parafollicular cell (C cells), which produce calcitonin and has a neuroendocrinal histogenesis [5]. An overview of MCT is discussed under Table 1.

Two different forms of MCT are recognized: the sporadic form, which accounts for about 75% of cases, and the hereditary or familial form accounting for the remaining 25%. RET (REarranged during Transfection) proto-oncogene mutation is identified on hot spots in most of the hereditary cases of MTC and less than half the cases of sporadic forms. RET tyrosine kinase receptor like other tyrosine kinases is involved in the regulation of differentiation, proliferation, survival, and cell motility processes through several intracellular signalling and hence plays a major role in histogenesis and evolution of MCT [6].

In recent years there has been gradual advancement of identification of more mutations like RAS in the so called RET negative cases. There is newer stress on reclassifying cases of MCT on the basis of molecular biology (Table 2). These along with a novel mutated form M918T are associated with more aggressive disseminated forms and presence of MCT in children including those seen in the present case of dermatological metastases (which are thought to carry some additional mutations) and carry poor prognosis [7, 8].

The newer research queers the pitch further by finding of an additional RAS mutations in all so called RET negative patients. One of the study has calculated the prevalence of RAS positivity in the range of 68% in RET negative MCT and only 2.8% in RET positive MCT [9] suggesting that RAS mutations could represent alternative genetic events in sporadic MCT tumorigenesis.

Thus a dermatologist/treating physician and pathologists should be aware of genetic classification of MTC (Table 2) in setting of dermatological metastases or other multicentric metastases in sporadic cases of young as various investigations and tools for genetic screening of patients and their relatives are now available which can identify germ line RET as well as other non-RET mutations. This in turn has helped treating physicians worldwide to understand the disease pathology, to tailor make the therapeutic response, and to frame recommendations in setting of MCT which can have a direct impact on disease free survival and can lessen the associated morbidity as well.

According to the latest recommendations it is encouraged that once an RET mutation has been confirmed in a patient, all first degree relatives should be screened to identify 50% who must have inherited the mutation (especially children) and are therefore at risk for development of MCT. All such patients can undergo prophylactic thyroidectomy [10] (Table 1). The newer research goes a step further with investigation a patient of non-RET mutation to investigate for other mutations such as RAS [11]. This approach has led to availability of newer therapeutic strategies involving newer tyrosine kinase inhibitors such as vandetanib and cabozantinib which are the drugs currently approved by US food and drug administration (FDA) for treatment of metastatic MCT. Till date no case has been documented which has utilized above two drugs to treat cutaneous metastasis [12, 13] for all such cases which are more complicated, undergone wide metastases (including dermatological), and patients having novel mutations. It is imperative to note though that till date no Ras-targeted therapies have been successful in these cases but have met with a little success in setting of aggressive mutation M918T [8] (Table 2). However, the documentation of such mutations in MCT may lead to designing of drug particularly targeting these mutations in near future.

A conclusive diagnosis of cutaneous metastasis of MCT usually requires a high index of suspicion and histopathological backing with immunohistochemical analysis. Apart from the characteristic “neuroendocrinal” morphology noted on cytology and histology, MCT is immunopositive for markers such as calcitonin (most specific tumour marker), synaptophysin, chromogranin, and CD56.

These arrays of IHC markers which are positive for neuroendocrinal tumours can be seen in other tumours such as small cell (SC) carcinoma lung, carcinoid tumours (of thoracic, abdominal, and head-neck region), and even merkel cell carcinoma. All these can also present as subcutaneous chest and scalp swellings. Here a unique and novel IHC marker, thyroid transcription factor (TTF-1), can be a very effective tool in distinguishing the type of malignancy [11]. TTF-1 is expressed in thyroid follicular cells, thyroid C cells, and pneumocytes. So it can effectively rule out carcinoid tumors/other neuroendocrinal tumors of extra-thyroid sites and hence narrowing the differentials to MCT and SC carcinoma lung.

The differentiation between MCT and SC carcinoma lung (as both express TTF-1 and neuroendocrinal markers) can be easily done on the basis of expression of thyroglobulin which is negative in the latter [14].

The tumour cells in the present case were immunoreactive to calcitonin, TTF-1, and thyroglobulin. It was immunonegative for S-100 which ruled out the possibility of subcutaneous amelanotic melanoma. Hence a final diagnosis of cutaneous metastasis of MCT was reported.

The cutaneous metastasis of thyroid carcinoma is a sign of dissemination and reflects a very poor prognosis (mean survival rate of 7–19 months) [4]. The residual tumour tissue metastasis can be measured by PET-CT scan by identifying the hypermetabolic foci in the skin lesions. Conventionally as illustrated in Table 1, the management of MCT includes surgery (alone, when condition detected early) and if high risk of regional metastasis is suspected then surgery is supplanted with radiotherapy. Unlike the other variants of thyroid malignancy, there is no role of radioiodine treatment in cases of MCT because of difference in histogenesis. Due to small number of MCT cases with skin metastasis the biological behaviour of tumour to various modalities has not been studied in detail. Till date, combination of radiotherapy (RT) and chemotherapy (CT) or CT alone have been tried with 50% success rate [4, 15]. The response to CT is predicted by estimating serum calcitonin and CEA levels. Another proposed clinical indicator, calcitonin doubling time (CDT), has been proposed which better predicts MCT survival and prognosis [13, 16].

As described above the newer modalities which are targeting the tyrosine kinase proteins (involved in growth of medullary cancer cells) have opened a new vista in the treatment of MCT. These have utilized the principle of molecular genetics involved in tumorogenesis of MCT.

The present case report is worth reporting as it not only presents a rare presentation of secondary cutaneous metastasis of MCT but also illustrates instance of careful clinical assessment while evaluating a case of skin nodule showing spindloid/plasmacytoid cytology. So clinically, the investigation of a flesh coloured subcutaneous nodule, presenting with a short duration, particularly in scalp, jaw, or anterior chest wall, should include possibility of metastatic deposits.

A dermatologist should be clinically aware of the possibility of an internal organ malignancy in patients with such presentation and ready to think “out of box” and get all the necessary investigations including utilizing the technologies such as FNAC, IHC, and molecular studies to identify gene mutations for proper evaluation. These principles were employed in the present case report to reach a diagnosis as diagnosis of MCT was not suspected clinically at its primary presentation.

A prompt and an emphatic diagnosis and treatment will have its bearing on the eventual outcome in all these patients.

## Figures and Tables

**Table 1:** Medullary carcinoma thyroid: an overview.

*Clinical examination *	
(i) Incidence	
3.0% of all thyroid cancers	
(ii) Age at presentation	
5th and 6th decade	
(iii) Clinical presentation at diagnosis	
(a) Cervical swelling (cervical lymphadenopathy) with midline neck swelling	
(b) Hoarseness, dysphagia, and stridor	
(c) Paraneoplastic syndromes (uncommon)	
(d) Diarrhoea	
(iv) Propensity for regional and distant metastasis	
(a) Cervical Lymphadenopathy present in 50% cases at the time of diagnosis	
(b) Liver, lung, and bone metastasis by hematogenous route in 5–10% cases at the time of diagnosis	

*Diagnostic options *	
(i) Cytology	
(ii) Histopathology followed by immunohistochemical stains	
(iii) Serum calcitonin and CEA levels	
(iv) 24 hours urinalysis for catecholamine metabolites to rule out asso MEN 2 syndrome	
(v) Radiological assessment	
(a) Whole body CT scan	
(b) Ultrasonography of neck and abdomen	
(vi) Screening for missense mutation in RET in leucocytes	

*Management options *	
(i) Surgery	
(a) Total thyroidectomy with or without neck dissection	
(b) Prophylactic thyroidectomy in carriers	
(ii) Radiotherapy (adjuvant)	
(iii) Chemotherapy (palliative in advanced cases)	
(iv) Newer modalities (tyrosine kinase inhibitors)	
(a) Vandetanib	
(b) Cabozantinib	

**Table 2:** Molecular classification of MCT (Modified from 2012 Europen thyroid cancer association guidelines and with work done by Boichard et al. [8]).

Hereditary MTC (25% Cases): Associated with almost all cases with germline RET mutation (Exon 5, 8, 10, 11, 13, 14, 15 & 16).	
- MEN 2A: 85% cases mutation at Exon 11, codon 634	
Other mutations Exon 10 and 11, codon 609, 611, 618, 620	
- MEN 2B: Exon 16, codon 918 (Most common)	
- Disseminated and aggressive variant (commonly in children and young): M918T	
Sporadic MTC (75% of all cases):	
- RET positive group: 35% cases with somatic RET mutations	
- RET negative group:	
(1) Criteria: Negative for common germline mutations in Exon 5, 8, 10, 11, 13, 14, 15 & 16	
(2) Other Mutations to be identified:	
(i) RAS mutation—(Almost 80% of remaining RET negative cases)	
(ii) H-RAS (>50%): Exon 2, codon 13; Exon 3, codon 61; Exon 4, codon 63	
(iii) K-RAS (<30%): Exon 3, codon 61; Exon 2, codon 13; Exon 4, codon 117	
